# Bioengineering of rFVIIa Biopharmaceutical and Structure Characterization for Biosimilarity Assessment

**DOI:** 10.3390/bioengineering5010007

**Published:** 2018-01-19

**Authors:** Othman Montacir, Houda Montacir, Murat Eravci, Andreas Springer, Stephan Hinderlich, Fereidoun Mahboudi, Amirhossein Saadati, Maria Kristina Parr

**Affiliations:** 1 Institute of Pharmacy, Department of Biology, Chemistry, Pharmacy, Freie Universität Berlin, Königin-Luise-Strasse 2+4, 14195 Berlin, Germany; montacir@zedat.fu-berlin.de (O.M.); houda@zedat.fu-berlin.de (H.M.); 2 Labor für Biochemie, Department of Life Sciences & Technology, Beuth Hochschule für Technik Berlin, Seestraße 64, 13347 Berlin, Germany; stephan.hinderlich@beuth-hochschule.de; 3 Institut für Chemie and Biochemie, Department of Biology, Chemistry, Pharmacy, Freie Universität Berlin, Takustrasse 3, 14195 Berlin, Germany; eravci@zedat.fu-berlin.de; 4 Core Facility BioSupraMol, Department of Biology, Chemistry, Pharmacy, Freie Universität Berlin, Takustrasse 3, 14195 Berlin, Germany; andreas.springer@fu-berlin.de; 5 AryoGen Pharmed, No. 140, Cross Tajbakhsh Street, 24th Kilometer Makhsous Road, Tehran, Iran; mahboudif@cinnagen.com; 6 Biopharmaceutical Research Center, AryoGen Pharmed Inc., Alborz University of Medical Sciences, Karaj, Iran; saadatirada@aryogen.com

**Keywords:** biopharmaceutical, biosimilar, mass spectrometry, physicochemical characterization, coagulation assay

## Abstract

Eptacog alfa (NovoSeven^®^) is a vitamin K-dependent recombinant Factor VIIa produced by genetic engineering from baby hamster kidney (BHK) cells as a single peptide chain of 406 residues. After activation, it consists of a light chain (LC) of 152 amino and a heavy chain (HC) of 254 amino acids. Recombinant FVIIa undergoes many post-translational modifications (PTMs). The first ten glutamic acids of the N-terminal moiety are γ-carboxylated, Asn145 and Asn322 are N-glycosylated, and Ser52 and Ser60 are O-glycosylated. A head-to-head biosimilarity study was conducted for the originator and the first biosimilar AryoSeven™ to evaluate comparable bioengineering. Physicochemical properties were analyzed based on mass spectrometry, including intact mass, PTMs and higher-order structure. Both biotherapeutics exhibit a batch-to-batch variability in their N-glycan profiles. N-Glycopeptide analysis with UHPLC-QTOF-MS^E^ confirmed N-glycosylation sites as well as two different O-glycopeptide sites. Ser60 was found to be O-fucosylated and Ser52 had O-glucose or O-glucose-(xylose)_1,2_ motifs as glycan variants. Ion mobility spectrometry (TWIMS) and NMR spectroscopy data affirm close similarity of the higher-order structure of both biologicals. Potency of the biodrugs was analyzed by a coagulation assay demonstrating comparable bioactivity. Consequently, careful process optimization led to a stable production process of the biopharmaceuticals.

## 1. Introduction

One of the major classes of therapeutical biopharmaceuticals are blood factors. They represent six percent of the approved biotherapeuticals up to 2014 [1]. Many of them are used to treat hemophilia, a hereditary (chromosome X) blood-coagulation disorder, due to a deficiency of the clotting factors VIII (hemophilia A) and IX (hemophilia B), respectively [2]. Activated factor VII (FVIIa) initiates the extrinsic coagulation pathway and thereby activates factor IX and factor X to bind to tissue factor on the surface of cells that were exposed to circulating blood due to an injury. The application of pharmacologic doses of FVIIa results in the binding of sufficient amounts of FVIIa to activated platelets and subsequent activation of factor X, further passing of the tenase complex and finally an inducing of thrombin burst [3].

Eptacog alfa is a vitamin K-dependent recombinant FVIIa (rFVIIa) produced by genetic engineering from baby hamster kidney (BHK) cells. The cleavage of Arg152–Ile153 bond of the single peptide chain of 406 residues (about 50 kDa) in FVII leads to its activation. The resulting FVIIa consists of a light chain (LC) of 152 amino acids (about 20 kDa) and a heavy chain (HC) of 254 amino acids (about 30 kDa), linked to each other by a single disulfide bond (Cys135–Cys262). During bioengineering rFVIIa undergoes many post-translational modifications (PTMs). The first ten glutamic acids of the N-terminal moiety are γ-carboxylated, Asn145 and Asn322 are N-glycosylated, and Ser52 and Ser60 are O-glycosylated. The N-glycosylation plays important roles in protein folding of FVII, while O-glycosylation is more meaningful for the biological activity of FVII. It was suggested that structural elements found in the O-glycans are important for the association of FVIIa with tissue factor [4,5]. γ-Carboxylation is described to be sensitive to limitations in the cellular post-translational protein modification machinery in the secretion pathway of recombinant vitamin K dependent coagulation factors [6].

Eptacog alfa with its trade name NovoSeven^®^ (Novo Nordisk, Bagsværd, Denmark) was regulatory licensed in 1996 (EU), 1999 (USA) and 2000 (Japan) to treat congenital and acquired hemophilia [7]. AryoSeven™ is a biosimilar version of NovoSeven^®^, which has been recently proved to be comparable in terms of clinical efficacy [8]. It has been designed by bioengineering to meet criteria of biosimilarity and clinical data are already available to show similar efficacy in humans [8]. In general, biosimilars represent a subclass of biotherapeutics with high structural and clinical similarities compared to the already marketed inventor biodrug [9]. According to the European Medicines Agency’s (EMA) biosimilar application pathway criteria or equivalent criteria from other countries, similarity of two biologicals needs to be demonstrated. Critical quality attributes (CQA) that may impact pharmacological response need to be investigated and specifications need to be set [10]. Since the variability of biological production processes may alter the product quality, batch-to-batch changes of CQA over an extended manufacturing period need to be thoroughly controlled. Consequently, the preset CQA specifications of the originator have to be realized in an acceptable range by engineering of the bioprocess for originator as well as for biosimilar production [11]. Several state-of-the-art analytics need to be applied to evaluate the requirement of International Conference on Harmonization (ICH) Q6B and Q5E specifications. They request an in-depth characterization and comparability of multiple CQA, concerning the physical, chemical and biological characteristics for at least three batches [12,13,14]. Since glycosylation is highly sensitive to manufacturing changes, EMA and Food and Drug Administration (FDA) request extensive glycoanalytics, including glycan pattern and site-specific glycosylation analysis for glycoprotein-based pharmaceutics approval. Alteration of glycosylation profiles and modifications in a biosimilar can hamper the approval as a follow-on biodrug due to the known impact on activity and pharmacokinetics [15,16,17]. Bioengineering of a follow-on rFVIIa biopharmaceutical was therefore performed to obtain a product that meets criteria for approval as biosimilar. A head-to-head biosimilarity study was applied for NovoSeven^®^ (originator) and AryoSeven™ (follow-on product). Physicochemical properties were analyzed based on mass spectrometry, including intact mass, PTMs and higher order structure (HOS), and other biophysical methods. This was complemented by a potency assay for coagulation to investigate potential influence of CQA variations on bioactivity.

Eptacog alfa with its trade name NovoSeven^®^ (Novo Nordisk, Bagsværd, Denmark) was regulatory licensed in 1996 (EU), 1999 (USA) and 2000 (Japan) to treat congenital and acquired hemophilia [7]. AryoSeven™ is a biosimilar version of NovoSeven^®^, which has been recently proved to be comparable in terms of clinical efficacy [8]. It has been designed by bioengineering to meet criteria of biosimilarity and clinical data are already available to show similar efficacy in humans [8]. In general, biosimilars represent a subclass of biotherapeutics with high structural and clinical similarities compared to the already marketed inventor biodrug [9]. According to the European Medicines Agency’s (EMA) biosimilar application pathway criteria or equivalent criteria from other countries, similarity of two biologicals needs to be demonstrated. Critical quality attributes (CQA) that may impact pharmacological response need to be investigated and specifications need to be set [10]. Since the variability of biological production processes may alter the product quality, batch-to-batch changes of CQA over an extended manufacturing period need to be thoroughly controlled. Consequently, the preset CQA specifications of the originator have to be realized in an acceptable range by engineering of the bioprocess for originator as well as for biosimilar production [11]. Several state-of-the-art analytics need to be applied to evaluate the requirement of International Conference on Harmonization (ICH) Q6B and Q5E specifications. They request an in-depth characterization and comparability of multiple CQA, concerning the physical, chemical and biological characteristics for at least three batches [12,13,14]. Since glycosylation is highly sensitive to manufacturing changes, EMA and Food and Drug Administration (FDA) request extensive glycoanalytics, including glycan pattern and site-specific glycosylation analysis for glycoprotein-based pharmaceutics approval. Alteration of glycosylation profiles and modifications in a biosimilar can hamper the approval as a follow-on biodrug due to the known impact on activity and pharmacokinetics [15,16,17]. Bioengineering of a follow-on rFVIIa biopharmaceutical was therefore performed to obtain a product that meets criteria for approval as biosimilar. A head-to-head biosimilarity study was applied for NovoSeven^®^ (originator) and AryoSeven™ (follow-on product). Physicochemical properties were analyzed based on mass spectrometry, including intact mass, PTMs and higher order structure (HOS), and other biophysical methods. This was complemented by a potency assay for coagulation to investigate potential influence of CQA variations on bioactivity.

Eptacog alfa with its trade name NovoSeven^®^ (Novo Nordisk, Bagsværd, Denmark) was regulatory licensed in 1996 (EU), 1999 (USA) and 2000 (Japan) to treat congenital and acquired hemophilia [7]. AryoSeven™ is a biosimilar version of NovoSeven^®^, which has been recently proved to be comparable in terms of clinical efficacy [8]. It has been designed by bioengineering to meet criteria of biosimilarity and clinical data are already available to show similar efficacy in humans [8]. In general, biosimilars represent a subclass of biotherapeutics with high structural and clinical similarities compared to the already marketed inventor biodrug [9]. According to the European Medicines Agency’s (EMA) biosimilar application pathway criteria or equivalent criteria from other countries, similarity of two biologicals needs to be demonstrated. Critical quality attributes (CQA) that may impact pharmacological response need to be investigated and specifications need to be set [10]. Since the variability of biological production processes may alter the product quality, batch-to-batch changes of CQA over an extended manufacturing period need to be thoroughly controlled. Consequently, the preset CQA specifications of the originator have to be realized in an acceptable range by engineering of the bioprocess for originator as well as for biosimilar production [11]. Several state-of-the-art analytics need to be applied to evaluate the requirement of International Conference on Harmonization (ICH) Q6B and Q5E specifications. They request an in-depth characterization and comparability of multiple CQA, concerning the physical, chemical and biological characteristics for at least three batches [12,13,14]. Since glycosylation is highly sensitive to manufacturing changes, EMA and Food and Drug Administration (FDA) request extensive glycoanalytics, including glycan pattern and site-specific glycosylation analysis for glycoprotein-based pharmaceutics approval. Alteration of glycosylation profiles and modifications in a biosimilar can hamper the approval as a follow-on biodrug due to the known impact on activity and pharmacokinetics [15,16,17]. Bioengineering of a follow-on rFVIIa biopharmaceutical was therefore performed to obtain a product that meets criteria for approval as biosimilar. A head-to-head biosimilarity study was applied for NovoSeven^®^ (originator) and AryoSeven™ (follow-on product). Physicochemical properties were analyzed based on mass spectrometry, including intact mass, PTMs and higher order structure (HOS), and other biophysical methods. This was complemented by a potency assay for coagulation to investigate potential influence of CQA variations on bioactivity.

## 2. Materials and Methods

### 2.1. Chemicals and Consumables

All purchased chemicals were of highest purity. Diammonium hydrogen citrate, dithiothreitol (DTT), iodoacetamide (IAA), D_2_O, dextran ladder, super-dihydroxybenzoic acid (sDHB), sodium hydroxide, methyl iodide, ammonium bicarbonate, MS grade acetonitrile (ACN), formic acid (FA), and trifluoroacetic acid (TFA) were purchased from Sigma-Aldrich (Steinheim, Germany). Dimethyl sulfoxide, sodium iodide, 2-propanol and chloroform were purchased from Carl Roth (Karlsruhe, Germany). Ultrapure water was obtained from a Milli-Q water purifier (Millipore Corporation, Medford, MA, USA). Sequencing grade trypsin and PNGase F were purchased from Roche Applied Science (Mannheim, Germany). ZIC-HILIC kit was purchased from Merck (Darmstadt, Germany), Amicon Ultra-0.5 mL centrifugal filters from Merck (Darmstadt, Germany), Empore^®^ solid phase extraction (SPE) used for C18 stage tips from Sigma-Aldrich, and carbograph extract clean columns from Alltech (Deerfield, IL, USA). The National Institute for Biological Standards and Control standard ampoule (NIBSC, UK) was obtained and used as a reference standard. Factor VII deficient plasma (Hemosil, NY, USA), calibration plasma (Hemosil) and thromboplastin (Hemosil) were purchased in lyophilized form and reconstituted according to the manufacturer’s instructions. Factor diluent and reference emulsion were obtained from Hemosil as well.

The originator “NovoSeven^®^ 1.2 mg/mL” (Lot-No: CU60561, ES6S824, and ES6T726) was obtained from Novo Nordisk (Bagsværd, Denmark). The biosimilar “AryoSeven™ 1.2 mg/mL” (Lot-No: 9401043-a, 9401044-a, and 9401045-a) was received from AryoGen Pharmed (Tehran, Iran). Both biologicals were delivered as a lyophilized powder for reconstitution in a vial.

### 2.2. Instrumentation

Glycopeptide analysis using liquid chromatography accurate mass spectrometry (UHPLC-QTOF-MS) was performed on a Waters ACQUITY UPLC system coupled to a SYNAPT HDMS G2-S mass spectrometer (Waters; Manchester, UK) with a resolution of 18,000 and equipped with a BEH C18 (2.1 × 100 mm, 1.7 µm particle size, Waters, Manchester, UK) using H_2_O:FA (99.9:0.1, v:v) as mobile phase A and ACN:FA (99.9:0.1, v:v) as mobile phase B. The flow rate was set to 200 µL/min and a linear gradient from 5% B to 35% B in 35 min, 35% B to 50% B in 10 min followed by an isocratic flow at 80% B for 5 min was applied. QTOF mass calibration was performed using the singly charged ions produced by a 2 μg/μL sodium iodide solution in 2-propanol:water (50:50, v:v). Samples were ionized in positive electrospray ionization (ESI+) mode. The capillary voltage was at 2500 V, cone voltage at 50 V, and acquisition range was *m*/*z* 250–2000. In elevated energy scan mass spectrometry (MS^E^) experiments acquisition range was *m*/*z* 50–2000, trap collision energy 4 V and transfer collision energy from 15 to 45 V.

Ion mobility spectrometry (IMS) analyses were carried out using analogous conditions as described for UHPLC-QTOF-MS, with additional usage of the IMS option (UHPLC-IMS). IMS settings were as following: capillary voltage 2800 V, wave velocity 2000 m/s, wave height 1.0 V, transfer wave velocity 1968 m/s and transfer wave height 1.8 V. Data interpretation was performed using MassLynx 4.1 and Driftscope 2.1 (Waters; Manchester, UK).

Matrix-assisted laser desorption ionization time of flight mass spectrometry (MALDI-TOF-MS) of N- and O-glycans was performed on an Ultraflextreme mass spectrometer (Bruker Daltonics; Bremen, Germany) equipped with a smartbeam-IITM laser and a TOF/TOF MS facility (LIFTTM technology). Aliquots (0.5 µL) of the sample were mixed directly on a ground steel MALDI target plate (Bruker Daltonics) in a 1:1 ratio (v/v) with 10 mg/mL sDHB matrix dissolved in ACN:H_2_O (10:90, v:v) and allowed to dry at ambient temperature. Spectra were recorded from *m*/*z* 700 to 5000 (N-glycans) or *m*/*z* 200 to 3000 (O-glycans) in the reflectron positive ion mode. Acquisition was proceeded at 2000 Hz for 4000 shots (200 shots per step) using a random-walk algorithm and the ions were accelerated with 25 kV. External calibration for glycans was performed using a dextran ladder (Sigma). Data analysis was performed in FlexControl 3.4 (Bruker Daltonics) and spectra were evaluated using the GlycoPeakfinder (EuroCarbDB, European Bioinformatics Institute, Heidelberg, Germany) software. Assigned glycan structures were built with the GlycoWorkbench software (1.1 version, EuroCarbDB).

Intact mass of biotherapeutics was assessed by MALDI-TOF-MS. Protein standard II (Bruker Daltonics) was used as an external calibration standard. Aliquots (2 µL) of the sample solution were mixed with 2 μL of a 2% TFA solution. The matrix solution (2 µL) was added and the mixture was pipetted up and down until the crystallization starts. An aliquot (1 μL) of the crystal suspension was spotted and thoroughly mixed on the target. Ions were analyzed in the linear mode after acceleration at 20 kV and the laser energy was set to 50%. 

Nanoflow liquid chromatography electrospray ionization tandem mass spectrometry (nLC-ESI-MS/MS) analyses were performed on a Dionex Ultimate 3000 nanoLC (Thermo Fisher Scientific; Bremen, Germany) coupled to a LTQ Orbitrap Velos mass spectrometer (Thermo Fisher Scientific) using nESI+ ionization. Chromatographic separation was achieved on an in-house manufactured 250 mm fritless silica microcolumn with an inner diameter of 100 μm. Columns were packed with ReproSil-Pur C18-AQ 3 μm resin (Maisch GmbH; Ammerbuch-Entringen, Germany). A linear gradient from 5% B to 60% B in 90 min at flow rate of 350 nL/min for mobile phase A (H_2_O:FA, 99.9:0.1, v:v) and mobile phase B (ACN:FA, 99.9:0.1, v:v) was applied. Full scan MS spectra (from *m*/*z* 300–1700) as well as product ion spectra after fragmentation of the twenty most intense ions by collision-induced dissociation (CID) were recorded. Protein sequence identification and PTM analysis were performed using MaxQuant, version 1.3.0.5 (Max-Planck-Institute of Biochemistry; Martinsried, Germany).

### 2.3. Sample Processing

#### 2.3.1. Intact Mass Determination

rFVIIa samples were buffer exchanged in 30 kDa MWCO Amicon ultra centrifugal filter units and diluted to 1 μg/μL in H_2_O:FA (9.9:0.1, v:v). Then, 2,5-dihydroxy actetophenone (2,5-DHAP) matrix (7.6 mg) was dissolved in 375 µL ethanol mixed with 125 µL of a solution containing 18 mg/mL of diammonium hydrogen citrate (DAC) dissolved in water and used for the analysis of native rFVIIa.

#### 2.3.2. N- and O-Glycan Analysis

Aliquots (50 μg) of biologicals were dissolved in 100 μL of 50 mM ammonium bicarbonate (ABC) buffer. Reduction was performed with DTT (20 mM in 50 mM ABC buffer) within 45 min at 60 °C and alkylation with 20 mM of IAA within 30 min at room temperature in the dark. Subsequently, samples were digested with 2 U PNGase F enzyme in 50 µL ABC buffer for 4 h at 37°C to release total N-glycans. In contrast, the O-linked carbohydrates were cleaved using β-elimination performed as follows: Aliquots of 50 μg of protein were mixed with 10 µL of 200 mM DTT and the resulting mixture was incubated for 60 min at 60 °C. The solution was allowed to cool down at RT, after which 10 μL of a 200 mM IAA solution in water were added and incubated at RT for 45 min in the dark. Subsequently, 50 µL of aqueous ammonia (25% v:v) were added to the mixture and incubated at 50 °C overnight. Samples were dried under vacuum. Purification was achieved using a Carbograph SPE cartridge equilibrated with 3 × 400 µL of ACN:H_2_O:TFA (80:19.9:0.1, v:v:v), followed by H_2_O:TFA (99.9:0.1, v:v). Samples were acidified with 1% TFA to a final pH value < 4 and applied onto the column. Columns were washed with 3 × 400 µL of H_2_O:TFA (99.9:0.1, v:v) in order to desalt N-glycans. Glycans were then eluted with 3 × 400 µL of ACN:H_2_O:TFA (25:74.9:0.1, v:v:v). Eluates were dried under vacuum. The dried N-glycans were permethylated in dimethyl sulfoxide using sodium hydroxide and methyl iodide as described in literature [18]. The mixtures were subsequently incubated at room temperature for 1 h. Samples were mixed vigorously, then the aqueous phase was discarded. The organic phase was washed five times with water and then evaporated to dryness. The permethylated N-glycans were dissolved in 10 µL of ACN:H_2_O (75:25, v:v) and aliquots were analyzed by MALDI-TOF-MS. 

#### 2.3.3. Peptide Mapping

Aliquots (50 μg) of biologicals were dissolved in 100 μL of 50 mM ABC buffer. Reduction was performed with DTT (20 mM in 50 mM ABC buffer) for 45 min at 37 °C and alkylation with 20 mM of IAA for 30 min at room temperature in the dark. Subsequently, samples were digested with 2 U PNGase F enzyme in 50 µL ABC buffer for 4 h at 37 °C. Deglycosylated rFVIIa samples were digested with trypsin (1 μg trypsin, 1:50 enzyme to protein ratio in 50 mM ABC buffer) and desalted using C18 stage tips. C18 tips were equilibrated with 100 µL MeOH, 190 µL of ACN:H_2_O:FA (5:94.9:0.1, v:v:v), followed by 120 µL ACN:H_2_O:TFA (5:92:3, v:v:v). Samples were applied and C18 tips were washed with 200 µL of ACN:H_2_O:FA (5:94.9:0.1, v:v:v). Peptides were then eluted with 200 µL of ACN:H_2_O:FA (80:19.9:0.1, v:v:v). Eluates were dried under vacuum. Purified peptide samples were dissolved in 15 µL of ACN:H_2_O:FA (5:94.9:0.1, v:v:v) and subjected to protein sequencing and PTMs analysis using nLC-ESI-MS/MS. Data were compared to DrugBank online database containing both HC and LC sequences of rFVIIa.

#### 2.3.4. Glycopeptide Analysis

Aliquots of the samples containing 50 μg of rFVIIa were dissolved in 100 μL of ABC buffer. Reduction was performed with DTT (20 mM in 50 mM ABC buffer) within 45 min at 60 °C and alkylation with 20 mM IAA within 30 min at room temperature in the dark. Trypsin (1 μg, 1:50 enzyme to protein ratio) was added and incubation was carried out over night at 37 °C. Prior to analysis, separation of the glycopeptides from hydrophobic peptides was performed using ZIC-HILIC ProteoExtract kit (Merck, Darmstadt, Germany) as described by the manufacturer. The resulting supernatant containing glycopeptides was analyzed by UHPLC-QTOF-MS. 

#### 2.3.5. Analysis of Higher Order Structure

rFVIIa samples were buffer exchanged into ABC buffer with a 30 kDa Amicon filter (Merck, Germany). rFVIIa samples reconstituted in 50 μL ABC buffer were delivered to the UHPLC-IMS and analyzed as described above. 

Secondary structures of innovator and biosimilar were compared by circular dichroism (CD) using a JASCO J-810 CD spectrometer (Jasco, Easton, MD, USA) equipped with 0.1 cm path length CD Suprasil cuvettes (Hellma Analytics, Müllheim, Germany). The analyses were carried out at 293 K. A band width of 2 nm and a scanning speed of 100 nm/min were used. Three scans were performed for each sample three times (*n* = 3), and the resulting spectra were averaged. Fourier transform infrared spectroscopy (FTIR) measurements were performed using a PerkinElmer Spectrum 100 FTIR spectrometer. The measurement time was set to 50 s with a resolution of 4 cm^−1^. 

One-dimensional proton NMR spectra were recorded on a Avance III 700 MHz NMR system (Bruker Daltonics) with Topspin 2.1 software. The formulation buffer of the biopharmaceuticals was changed to water using a 30 kDa Amicon filter. Heavy water was used to suppress the residual water signal. The measurement of samples at concentrations of 0.25 mM were performed with a spectral width of 20 ppm and 8k scans at 293 K.

#### 2.3.6. Coagulation Assay

FVIIa activity was analyzed by automatic coagulation analyzer, ACL 7000 (Instrumentation Laboratory, Bedford, MA, USA). The ACL 7000 methodology is based on the change of light scatter associated with the formation of a fibrin clot. After calibration of the instrument, the total content of NIBSC standard ampoule, containing the 2nd International Standard for FVII concentrate, was reconstituted and serial dilutions of standard and sample were prepared according to the manufacturers’ instructions. Clotting times for each dilution were analyzed by the PLA 2.0 Bioassay software (Stegmann Systems, Rodgau, Germany). Average results of the three purchased NovoSeven^®^ originator lots (Lot-No: CU60561, ES6S824, ES6T726) were used as a reference value.

## 3. Results

Two rFVIIa biotherapeuticals were analyzed to achieve physicochemical characterization and comparison of their properties. AryoSeven™ is considered as follow-on biopharmaceutical. Bioengineering was performed to obtain a product that meets criteria for approval as biosimilar. A head-to-head biosimilarity study was performed using the originator NovoSeven^®^. Batch-to-batch variabilities were monitored in both products to evaluate CQA specifications. Intact mass, PTMs including glycosylation, and higher order structure were analyzed. 

### 3.1. Intact Mass

**Figure 1:**
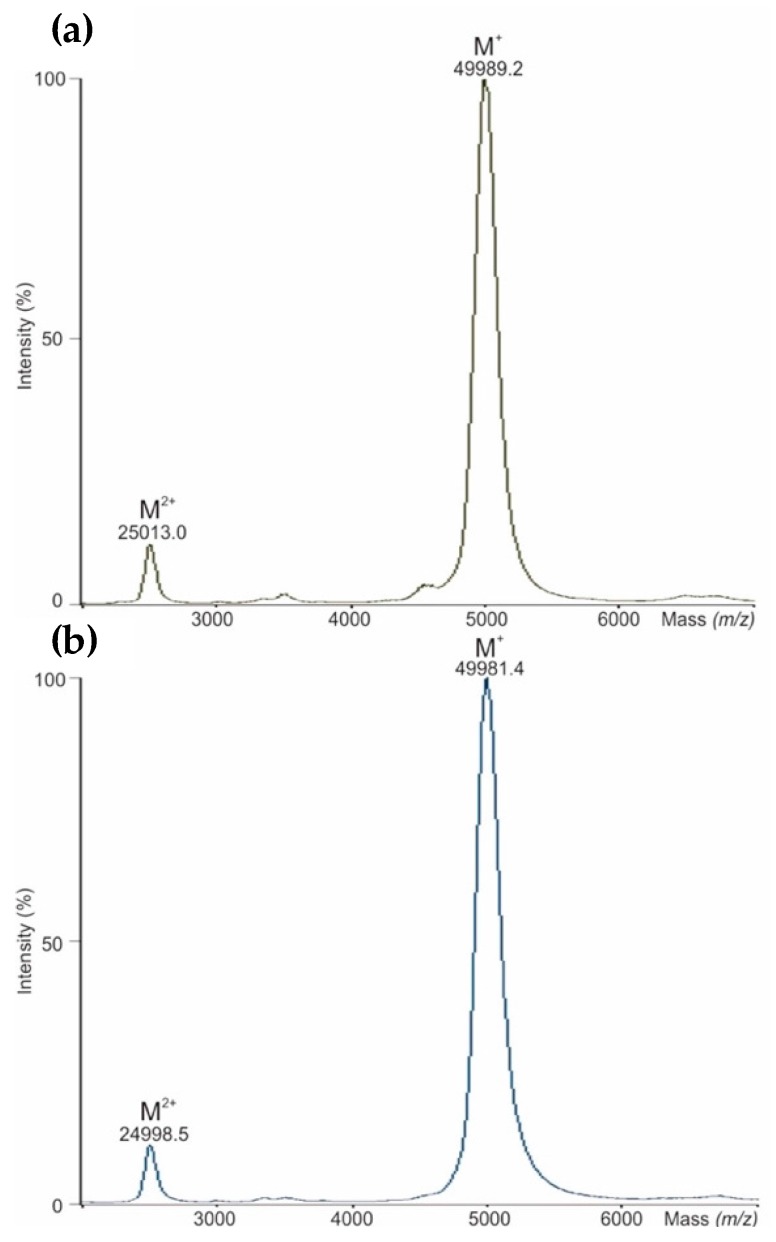
(**a**) Matrix-assisted laser desorption ionization time of flight mass spectrometry (MALDI-TOF-MS) analysis of intact mass for NovoSeven^®^, and (**b**) for AryoSeven™.

The intact mass measurements of a reference product (NovoSeven^®^) and its bioengineered biological (AryoSeven™) were performed by means of MALDI-TOF-MS after a desalting step. Two broad signals were determined, due to PTMs such as glycosylation and γ-carboxylation, presenting singly [M+H]^+^ and doubly [M+2H]^2+^ charged molecules (Figure 1). Head-to-head comparison exhibits the similarity of the molecular masses of both biologicals. However, a mass shift of about eight daltons for NovoSeven^®^ was observed. 

### 3.2. PTMs

Bottom-up approach is a useful implement to investigate the amino acid (AA) sequence and to identify PTMs from manufacturing process. Originator and its follow-on biologic were tryptically digested and resulting peptides were introduced to nLC-ESI–MS/MS. The resulting peptide fragment data were searched using MaxQuant to confirm the primary sequence and PTMs. The investigation of peptide maps resulting from tryptic digestion show high similarity and identical AA sequence, confirming the full truncation of propeptide AA sequence. PTMs in NovoSeven^®^ and AryoSeven™ were further investigated and compared (Table 1). 

A similar degree of oxidation and deamidation was detected in both biotherapeutics. The deamidation and oxidation are very common in protein therapeutics. During oxidation, a sulfur residue at methionine is oxidized into a sulfoxide (mass increase of 16 Da) and subsequently into a sulfone form (mass increase of 32 Da). During deamidation Asn residues are converted to Asp and/or isoAsp (mass increase of 1 Da). However, all peptides containing these modifications were minor in comparison to the same peptides without modification, indicating that the modifications were present at low levels in the molecules. On average, the number and locations of the modifications found in the follow-on and originator biotherapeutic were comparable (Table 1).

**Figure 2:**
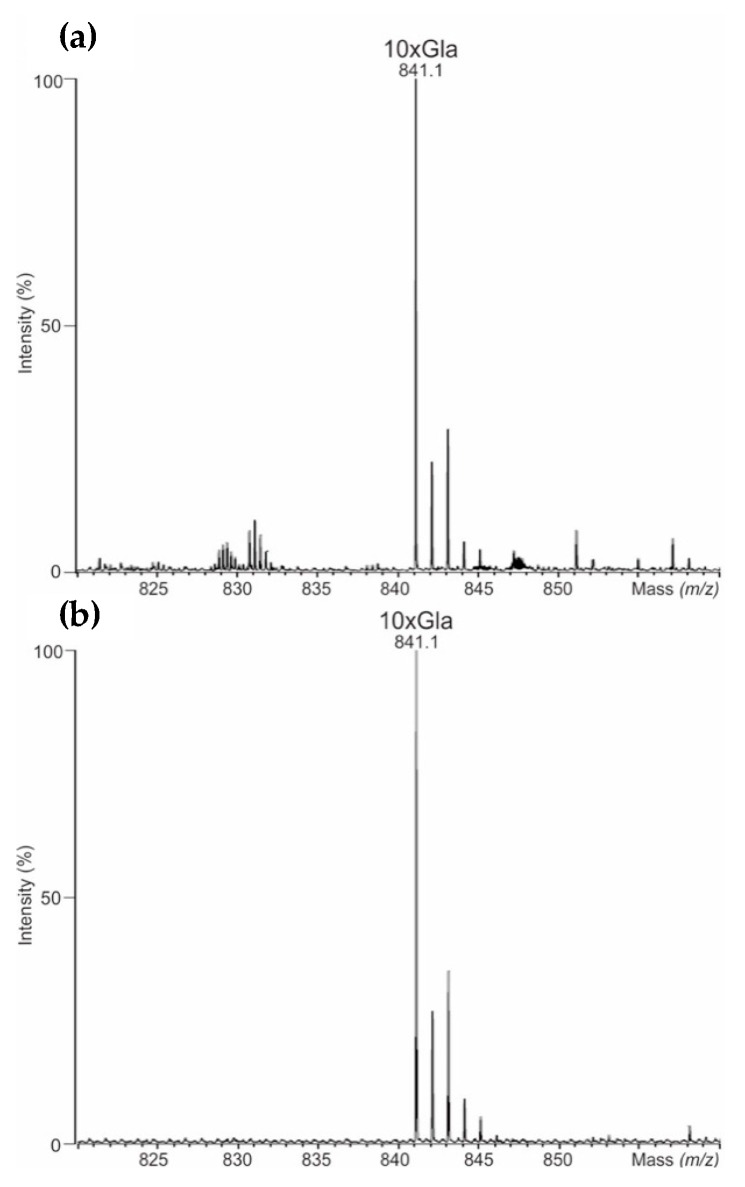
(**a**) Mass spectra of γ-carboxylation detected using liquid chromatography accurate mass spectrometry (UHPLC-QTOF-MS)/MS^E^ in NovoSeven^®^, and (**b**) AryoSeven™. Not assigned signals were artifacts.

Furthermore, the γ-carboxylation in all rFVII preparations of NovoSeven^®^ and AryoSeven™ were investigated. LC-QTOF-MS of the tryptic fragments representing the Gla domain confirmed the existence of fully modified (10-times Gla) as six-fold protonated peptide *m*/*z* 841.3 (theoretical mass 5044.1 Da) in originator as well as follow-on biologic (Figure 2). Signals of the six-fold protonated peptides with 9-times Gla (*m*/*z* 833.4) and 8-times Gla (*m*/*z* 826.0) were not detected, indicating complete γ-carboxylation of both biologics.

### 3.3. Glycosylation

Characterization of glycosylation sites in medicinal products is one of the major requirements of health care regulatory bodies. Alterations in the bioprocess may cause changes in glycosylation. Bioprocess engineering is therefore of particular interest. Subsequent glycosylation analysis can be a challenging task regarding the numerous N- and O-glycosylation sites and the multiple glycan pattern of rFVIIa due to macro- and microheterogeneity. 

#### 3.3.1. Glycopeptide Analysis

**Figure 3:**
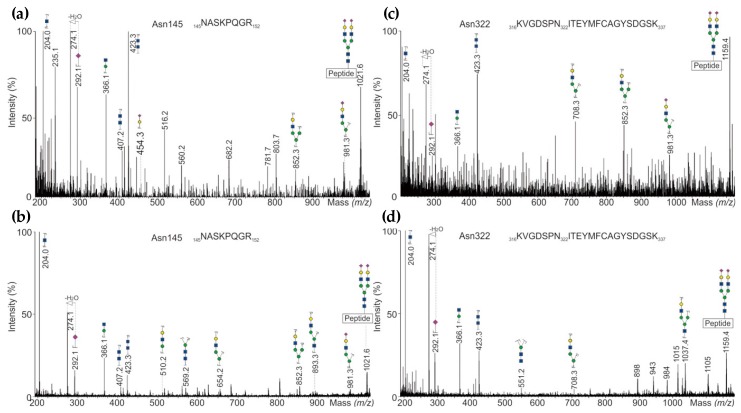
(**a**) UHPLC-QTOF-MS/MS^E^ spectra of N-glycopeptides representing N-glycosylation site Asn145 detected in NovoSeven^®^, and (**b**) in AryoSeven™; (**c**) site Asn322 in NovoSeven^®^, and (**d**) in AryoSeven™.

Biotherapeutics were first reduced, alkylated and digested before introducing to ESI-MS. Mass spectra of tryptic glycopeptides obtained from LC-QTOF-MS^E^ analysis confirmed the presence of triply and quadruply charged ions at *m*/*z* 1021.4 and *m*/*z* 1158.6 corresponding to the most abundant glycoform of the tryptic glycopeptides including glycosylation sites at Asn145 (NASKPQGR) and Asn322 (KVGDSPNITEYMFCAGYSDGSK), respectively. The resulting fragment ions under CID conditions are exemplarily shown in Figure 3. Low-mass carbohydrate-specific oxonium ions such as *m*/*z* 204.1 for HexNAc^+^, *m*/*z* 274.1 and *m*/*z* 292.1 for N-acetylneuraminic acid (Neu5Ac)^+^, and *m*/*z* 366.1 for Hex-HexNAc^+^ were detected as diagnostic ions to assign the glycopeptides. Furthermore, the existence of characteristic carbohydrate ions at *m*/*z* 274.1 and *m*/*z* 292.1 confirmed that the glycopeptide is sialylated. In addition, a carbohydrate-specific oxonium ion corresponding to two HexNAc with *m*/*z* 423.1 was found in all MS^E^ spectra. 

**Figure 4:**
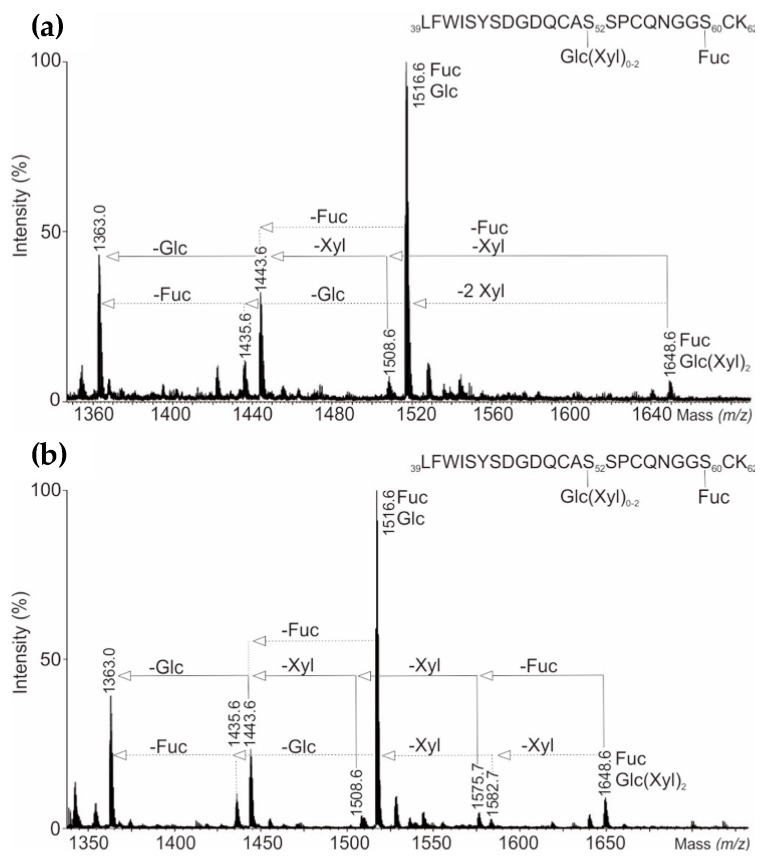
(**a**) Combined precursor ESI-QTOF low-energy scan mass spectra of doubly-charged O-glycopeptides from UHPLC-QTOF-MS/MS^E^ peptide mapping analysis showing O-glycosylation sites Ser52 and Ser60 of NovoSeven^®^, and (**b**) AryoSeven™.

Four O-glycopeptide signals within the doubly-charged precursor ions from summed chromatogram MS^E^ low energy data were further evaluated (Figure 4). They identified four different glycoforms of O-glycopeptides. The most abundant MS^E^ spectra of doubly-charged ion at *m*/*z* 1516.6 (LFWISYSDGDQCAS52SPCQNGGS60CK) was found to possess two O-glycosylation sites bearing glucose and fucose oligosaccharides on Ser52 and Ser60, respectively. However, the doubly-charged ion *m*/*z* 1648.6 represents a fully glycosylated peptide, bearing both, a fucose and glucose-(xylose)2 motif. Furthermore, low energy CID conditions enabled the detection of other glycopeptide isoforms such as doubly-charged ions *m*/*z* 1508.6 bearing glucose-xylose_1_ motif, *m*/*z* 1443.6 carrying one glucose, *m*/*z* 1435.6 with one fucose and aglycosylated peptide *m*/*z* 1363.6. The O- and N-glycopeptide mass spectra of follow-on biological (AryoSeven™) and reference product (NovoSeven^®^) were comparable.

#### 3.3.2. N-Glycans

**Figure 5:**
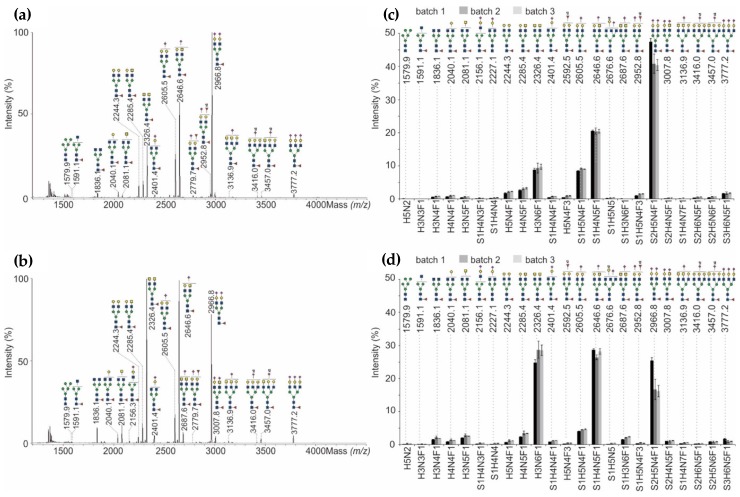
(**a**) MALDI-TOF-MS spectra of the most abundant permethylated N-glycans released from NovoSeven^®^, and (**b**) AryoSeven™. (**c**) Relative abundance of permethylated N-glycans from three batches of NovoSeven^®^, and (**d**) AryoSeven™ were calculated. Standard deviations were calculated from technical triplicates.

PNGase F-released N-glycans of three batches of originator and bioengineered follow-on product each were analyzed by means of MALDI-TOF mass spectrometry (Figure 5). The digested samples were purified and desalted, permethylated and analyzed using the positive ionization mode of MALDI-TOF-MS to ensure proper detection of neutral as well as sialylated glycan structures.

Within all batches of NovoSeven^®^, the biantenarry bigalactosylated N-glycan with one core-fucose (F) as well as two terminal Neu5Ac (=sialic acid, S), having the composition S2H5N4F1 (*m*/*z* 2966.8), was the most abundant structure. Second most abundant N-glycan was S1H4N5F1 at *m*/*z* 2646.6 (one galactose, one GalNAc and one terminal Neu5Ac), followed by S1H5N4F1 at *m*/*z* 2605.5 (two galactoses and one terminal Neu5Ac) and H3N6F1 at *m*/*z* 2326.4 (two terminal GalNAc). This shows that NovoSeven^®^ bears mainly biantenarry N-glycans modified with one fucose and/or one Neu5Ac residue (Figure 5d). The relative amounts of N-glycans within the three batches were almost comparable. Only the most abundant glycoform S2H5N4F1 showed a slightly higher abundance in batch one compared to batch two and three.

Comparing the originator and follow-on product, the data revealed that N-glycan patterns are qualitatively similar, but quantitatively heterogeneous. The overall distribution of the four most abundant structures (*m*/*z* 2326.4, 2605.5, 2646.6 and 2966.8) with a total abundance of about 80% was not identical for both biologicals. AryoSeven™ N-glycan profiles showed different order of abundances. The major structures were S1H4N5F1 (*m*/*z* 2646.6) and H3N6F1 (*m*/*z* 2326.4), followed by S2H5N4F1 (*m*/*z* 2966.8) and S1H5N4F1 (*m*/*z* 2605.5). Only minor quantitative variances mainly in the relative amounts of the compositions S2H5N4F1, S1H4N5F1 and H3N6F1 were detected when comparing batch one of the follow-on biologic to batch two and three. 

The most relevant difference between originator and follow-on product was observed with an excess of terminal GalNAc residues in the follow-on product samples, which correlated with an overall lower sialylation. However, batch-to-batch variances were minor for almost all structures except for batch one in the originator and biosimilar rFVIIa. Besides biantenarry complex-type N-glycans, rFVIIa biotherapeutics were shown to contain a comparable amount of high-mannose (H5N2 at *m*/*z* 1579.6). The neutral N-glycans were shown to be slightly increased in the biosimilar. Furthermore, triantennary species (S1H4N7F1 at *m*/*z* 3136.9, S2H6N5F1 at *m*/*z* 3416.0, S2H5N6F1 at *m*/*z* 3457.0 and S3H6N5F1 at *m*/*z* 3777.2), which are mono-, di- and trisialylated, were detected in low level in both biologicals. However, the glycan pattern of originator contains more sialylated structures in comparison to the biosimilar. The follow-on biologic possesses mainly the non-sialylated structures ending with Gal and GalNAc (galacto- and agalacto-type), respectively. 

### 3.4. Higher Order Structure

The measurement of secondary, tertiary and quaternary structure of biotherapeutics is a major task in the biopharmaceuticals characterization, giving information about the three-dimensional structure and consequently about the function of the biologicals. However, the complexity of biomolecules due to physicochemical properties impose an analytical challenge. The orthogonal methods CD and FTIR were applied to investigate the secondary and tertiary structure of NovoSeven^®^ and AryoSeven™. 

**Figure 6:**
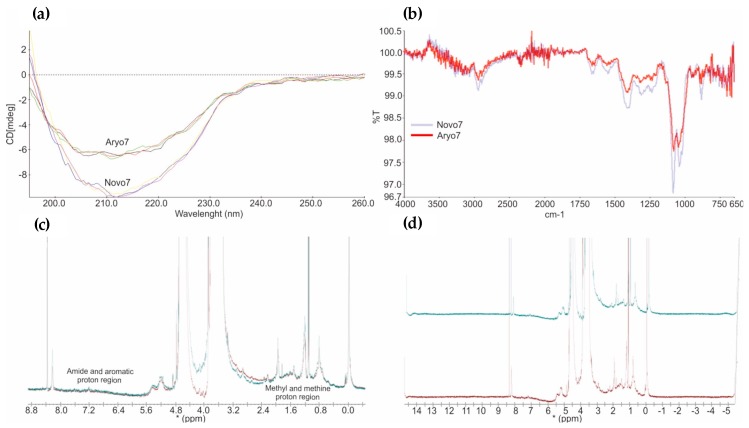
(**a**) The overlay of far-UV CD spectra obtained from innovator and AryoSeven™. (**b**) FTIR spectra indicating a high similarity of the amide I and II bands between both biologics (light blue; NovoSeven^®^, and red, AryoSeven™). (**c**,**d**) NMR spectral fingerprinting showing all protons in methyl and methine region (zero to three parts per million) as well as amide and aromatic region (six to eight parts per million) (green, NovoSeven^®,^ and red, AryoSeven™).

CD spectral analysis (Figure 6) in the far UV (200–260 nm) exhibit information about the α-helix, β-sheet and random coil present in the molecule, and near UV (260–350 nm) spectra represent the tertiary structure of proteins. The CD data (Figure 6a) revealed an absorbance minimum in the wavelength range of 190–230 nm, representing a predominant β-sheet secondary structure in both biotherapeutics. Homogeneous CD spectra in the range of 190–260 nm (Figure 6) confirmed the similarity of secondary structural elements of innovator and follow-on biological. Minor differences in CD spectra were observed, which were due to buffer variations only.

FTIR presents the vibrational state of biomolecules. Multiple IR bands (Figure 6b) are produced from the backbone amide groups, which describe the backbone shape of biologicals. A comparable result for both biotherapeuticals was obtained from FTIR technique. This shows similar site and shape of the amide bands (between 1750 and 750 cm^−1^) and confirmed the results from CD.

NMR spectroscopy was used for spectral fingerprint of the biologicals in solution. Protons in the methyl and methine region (zero to three parts per million) as well as protons in the amide and aromatic region (six to eight parts per million) showed similar resonance frequencies (Figure 6c,d) for originator and follow-on biological, which affirmed the similarity of their spatial shape. 

**Figure 7:**
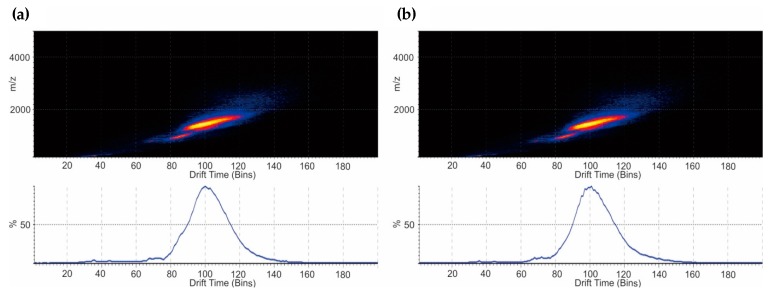
(**a**) The drift time generated from traveling wave IMS after IM-MS analysis under denaturing conditions for NovoSeven^®^; and (**b**) AryoSeven™ displayed homogenous driftscope plots between 60 and 140 Bins.

IMS allowed the illustration of conformational variations. Drift time analysis under denaturing conditions revealed similar traveling wave IMS (TWIMS) between NovoSeven^®^ and AryoSeven™ (Figure 7a,b). Highly charged species assembled homogeneous IMS plots at drift time between 60 and 160 Bins (Figure 7a,b).

### 3.5. Bioactivity

The potency of both rFVIIa biologicals was assigned by adding a thromboplastin reagent, that contains tissue factor and calcium, to FVII-deficient plasma, followed by measurement of the clotting time. Data were entered into PLA software to obtain potency ratio (Table 2), and showed that the potency ratio of biosimilar FVII was within 97 to 99% for all three batches, which is in the range of technical deviation of this assay. Bioactivity of the biosimilar could therefore be assigned as identical to the originator NovoSeven^®^. 

## 4. Discussion

Intact masses were found to be comparable for NovoSeven^®^ and AryoSeven™. The mass shifts observed for the biosimilar are most likely from variances in PTMs, such as deamidation [19], oxidation or glycosylation, which are closer evaluated after digestion of the protein. During the oxidation process, the sulfur atom at methionine is oxidized into a sulfoxide (mass increase of 16 Da) and subsequently into a sulfone form (mass increase of 32 Da) [20]. The process of deamidation occurs mainly at Asn residues either under acidic conditions or neutral and basic conditions leading to Asp and/or isoAsp with a mass increase of 1 Da [21,22]. A similar low degree of oxidation and deamidation was detected in both biotherapeutics, indicating that these modifications only have very low impact on the bioactivity. 

γ-Carboxylation was analyzed, since it was reported for monitoring of the limitations in the cellular post-translational protein modification machinery in the secretion pathway of recombinant vitamin K dependent coagulation factors [6,23]. The γ-carboxyglutamic acid-rich (GLA) domain in rFVII contains multiple glutamate residues (at E6, E7, E14, E16, E19, E20, E25, E26, E29, and E35) that have been post-translationally modified by vitamin K-dependent carboxylation to form γ-carboxyglutamate (Gla) [24,25,26]. γ-Carboxylated peptides were found to be comparable in NovoSeven^®^ and AryoSeven™ since fully γ-carboxylated peptide containing 10-times Gla was found in both biologics. Hence, this PTM is in line with previous studies [23]. 

Changes in glycosylation are often caused by alterations in the bioprocess used for production of biologicals [27,28]. In particular, O-glycosylation site identification is a challenging task, because Eptacog alfa bears O-fucosylation at position Ser60 and O-glucose or O-glucose-(xylose)_1,2_ motifs at Ser52 [29]. Since the glycosidic bond of O-fucose to Ser is highly labile, it causes complex CID mass spectra [30]. However, within this study the O- and N-glycopeptide mass spectra of follow-on biological (AryoSeven™) and reference product (NovoSeven^®^) were comparable and enabled to confirm each O- and N-glycosylation sites (Ser52, Ser60, Asn145 and Asn322). Thus, by carefully designing the bioengineering process it was possible to obtain biosimilarity also in this CQA. GalNAc-bearing N-glycan structures were found to be much more abundant in AryoSeven™, which is often correlated with an overall lower sialylation [23]. The latter one was also observed in the analyzed samples. The follow-on biologic possesses mainly the non-sialylated structures ending with Gal and GalNAc (galacto- and agalacto-type), respectively. Both structures bind to the asialoglycoprotein receptor with high affinity. This may accelerate clearance of rFVIIa from blood circulation and lead to a shortened half-life of a drug [31]. 

CD and FTIR were used to assess multiple geometries such as α-helices, polyproline helices, β-sheets and random coil, which impact the far ultraviolet CD spectrum and IR bands [13]. CD spectral analysis in the far UV (200–260 nm) exhibit information about the α-helix, β-sheet and random coil, which can resume the conformation state of the molecule. Near UV (260–350 nm) spectra represent the tertiary structure of proteins [32]. The CD data (Figure 6) revealed an absorbance minimum in the wavelength range of 190–230 nm, representing a predominant α-helical structure in both biotherapeutics [33]. Similar site and shape of the amide bands (between 1750 and 750 cm^−1^) in FTIR spectra confirmed the results from CD [13,32]. Minor differences in CD spectra arise from the variation of the calcium amount in the formulation buffer of the biologics [33], and did not reflect structural differences of the two proteins.

NMR spectroscopy can produce a spectral fingerprint of biologicals in solution, providing structural and hydrodynamic data for assessing biopharmaceuticals’ comparability [32,34]. NMR data were also found to be comparable for NovoSeven^®^ and AryoSeven™. 

Using IMS as a powerful visualization technique under denaturing conditions supported the biosimilarity study [35,36]. The separation of ion species with different size, charge and shape was performed in a drift cell filled with a buffer carrier gas (N_2_) in the presence of an electric field, where the speed of an ion species plays a critical parameter [37]. The data from IMS were highly comparable. 

The potency of both rFVIIa biologicals was assigned by adding a thromboplastin reagent that contains tissue factor and calcium to Factor VII-deficient plasma and measuring the clotting time. The prothrombin time (PT) varies with reagent and coagulometer, but typically ranges between 10 and 14 s [38]. The bioactivity of the biosimilar is almost identical to the originator NovoSeven^®^.

## 5. Conclusions

Bioengineering was successfully performed for the FVIIa follow-on biopharmaceutical AryoSeven™. In-depth characterization was performed using analytical methods such as MALDI-TOF/TOF-MS, UHPLC-QTOF-MS^E^, and IMS, and coagulation assay studies were applied to perform head-to-head comparison. Only small batch-to-batch variations were observed between three different batches of AryoSeven™ and for the originator NovoSeven^®^, which was analyzed in parallel. This demonstrates that careful process optimization led to a stable production process of the biopharmaceuticals. Furthermore, it was demonstrated that the follow-on biological was successfully designed as biosimilar, which was shown by comparable intact masses, PTMs (glycosylation and γ-carboxylation), higher order structure and potency characteristics. Intact mass analysis revealed a mass of apparently 50 kDa for originator and follow-on biological. The γ-carboxylation in all rFVIIa isolates of NovoSeven^®^ and AryoSeven™ were investigated. Ten times Gla-containing peptides were found in both biologicals. Glycosylation analysis revealed that the glycan pattern of originator contains more sialylated structures in comparison to the follow-on biologic. Its production mainly yielded the non-sialylated structures ending with Gal and GalNAc, which bind the asialoglycoprotein receptor with high affinity, suggesting an influence on half-life [31]. Higher degrees of sialylation were also reported to increase the half-life in vivo [39]. However, the clinical trial comparing both biotherapeuticals revealed the same efficiency in patients with hemophilia [8]. Thus, these differences may be considered of low relevance. 

## Figures and Tables

**Table 1:** Comparison of partial post-translational modification type and modified peptides found in NovoSeven^®^ and AryoSeven™ after tryptic digestion revealed by nanoflow liquid chromatography electrospray ionization tandem mass spectrometry (nLC-ESI–MS/MS; processed with MaxQuant).

Sequence	Length	Modifications	Modified Sequence
DDQLICVNENGGCEQYCSDHTGTKR	25	Asn->Asp	_DDQLICVNEN(as)GGCEQYCSDHTGTKR_
FSLVSGWGQLLDRGATALEL MVLNVPRLMTQDCLQQSR	38	Oxidation (M)	_FSLVSGWGQLLDRGATALELM VLNVPRLM(ox)TQDCLQQSR_
GATALELMVLNVPR	14	Oxidation (M), Asn->Asp	_GATALELM(ox)VLN(as)VPR_
GECPWQVLLLVNGAQLCGGTL INTIWVVSAAHCFDKIK	38	2 Asn->Asp	_GECPWQVLLLVN(as)GAQLCGGTL IN(as)TIWVVSAAHCFDKIK_
KVGDSPNITEYMFCAGYSDGSKDSCK	26	Oxidation (M), Asn->Asp	_KVGDSPN(as)ITEYM(ox)FCAGYSDGSKDSCK_
LFWISYSDGDQCASSPCQNGGSCK	24	Asn->Asp	_LFWISYSDGDQCASSPCQN(as)GGSCK_
LMRSEPRPGVLLR	13	Oxidation (M)	_LM(ox)RSEPRPGVLLR_
LMTQDCLQQSRK	12	Oxidation (M)	_LM(ox)TQDCLQQSRK_
NCETHKDDQLICVNENGG CEQYCSDHTGTKR	31	Asn->Asp	_NCETHKDDQLICVN(as)ENGG CEQYCSDHTGTKR_
NLIAVLGEHDLSEHDGDEQSRR	22	Asn->Asp	_N(as)LIAVLGEHDLSEHDGDEQSRR_
RVAQVIIPSTYVPGTTNHDIALLR	24	Asn->Asp	_RVAQVIIPSTYVPGTTN(as)HDIALLR_
VCPKGECPWQVLLLVNGAQ LCGGTLINTIWVVSAAHCFDK	40	Asn->Asp	_VCPKGECPWQVLLLVN(as)GAQ LCGGTLINTIWVVSAAHCFDK_
VGDSPNITEYMFCAGYSDGSKDSCK	25	Oxidation (M), Asn->Asp	_VGDSPN(as)ITEYM(ox)FCAGYSDGSKDSCK_

**Table 2:** AryoSeven™ biosimilar potency values measured by coagulation assay in comparison to the originator NovoSeven^®^. Assays were performed in triplicates.

Biosimilar Batch No.	Potency Ratio
9501047-a vs. Originator (NovoSeven^®^)	0.97
9501048-a vs. Originator (NovoSeven^®^)	0.99
9501050-a vs. Originator (NovoSeven^®^)	0.99
